# Trends of hip arthroscopy in Italy show an increase over the years: A nationwide study from 2001 to 2016

**DOI:** 10.1002/jeo2.70099

**Published:** 2024-12-19

**Authors:** Umile Giuseppe Longo, Rocco Papalia, Sergio De Salvatore, Giovanni Intermesoli, Ilaria Piergentili, Stefano Zaffagnini, Kristian Samuelsson, Pieter D'Hooghe, Vincenzo Denaro

**Affiliations:** ^1^ Department of Orthopaedics Fondazione Policlinico Universitario Campus Bio‐Medico Roma Italy; ^2^ Research Unit of Orthopaedic and Trauma Surgery, Department of Medicine and Surgery Università Campus Bio‐Medico di Roma Roma Italy; ^3^ Department of Orthopaedics IRCCS Ospedale Pediatrico Bambino Gesù Rome Italy; ^4^ Department of Biomathematics CNR‐IASI, Laboratorio di Biomatematica, Consiglio Nazionale delle Ricerche, Istituto di Analisi dei Sistemi ed Informatica Rome Italy; ^5^ Department of Orthopaedics Clinica Ortopedica e Traumatologica II IRCCS Istituto Ortopedico Rizzoli Bologna Bologna Italy; ^6^ Department of Orthopaedics Sahlgrenska Sports Medicine Center Gothenburg Sweden; ^7^ Department of Orthopaedics Institute of Clinical Sciences, Sahlgrenska Academy University of Gothenburg Gothenburg Sweden; ^8^ Department of Orthopaedics Sahlgrenska University Hospital Mölndal Sweden; ^9^ Department of Orthopaedics Aspetar Hospital Doha Qatar

**Keywords:** epidemiology, hip arthroscopy, Italy, registry, surgery

## Abstract

**Purpose:**

Hip arthroscopy has gained popularity among orthopaedic procedures over the last decade. The present study aims to evaluate the trends of hip arthroscopy procedures in Italy analysing data recorded by the Italian Ministry of Health, reporting demographics, length of hospitalisation (LOS), diagnosis and the economic impact of the procedure.

**Methods:**

The National Hospital Discharge records reported by the Italian Ministry of Health were used, analysing the data regarding hip arthroscopy procedures performed in Italy between 2001 and 2016. A total of 4022 hip arthroscopies were performed in Italy in the adult population.

**Results:**

The present study shows more males being exposed to the procedure, while the mean age of females was always higher than that of males during the 16‐year study period. The mean LOS was 2.6 ± 3.2 days, and every procedure had a mean Italian hospital reimbursement from 1361€ to 1512€. Hip arthroscopy procedures have increased substantially between the years 2001 and 2016, as well as the costs related to the surgery.

**Conclusions:**

The findings of this study constitute valuable clinical and scientific evidence for the healthcare system, which is useful not only for the formulation and improvement of national healthcare policies but also to improve awareness and accessibility of hip arthroscopy.

**Level of Evidence:**

Level II.

**Abbreviations**
ICD‐9‐CM: International Classification of Diseases, Ninth Revision, Clinical Modification
ISTAT: National Institute for Statistics
LOS: length of hospitalisation
SDO: National Hospital Discharge records

## INTRODUCTION

Hip arthroscopy is a minimally invasive surgical procedure that allows the diagnosis and treatment of intra‐articular hip pathology [7]. It was performed for the first time by Michael S. Burman in 1931, recording his first attempts on cadavers [10]. Access and manoeuverability of instrumentation are hindered by the unique anatomy of the hip, slowing the refinement of the technique [11]. Nevertheless, the introduction of new technologies and equipment has contributed to an increase in the application of hip arthroscopy, with an associated notable rise in the number of hip arthroscopies performed in recent years [17]. In particular, Bozic et al. [1] showed an increment of 600% in the overall incidence of hip arthroscopy procedures in the United States between 2006 and 2010. Arthroscopy performed by trained surgeons offers the advantages of an outpatient procedure and circumferential access to the hip joint and does not preclude the opportunity to perform future surgical interventions [7]. Nonetheless, treatment failures after surgery have been described [23]. Understanding complications and unsatisfactory outcomes is crucial to improve patient satisfaction, as well as details about nationwide epidemiological trends of hip arthroscopy and public health costs are essential in the formulation of healthcare and economic policies [13]. In Europe, three valid registries reporting epidemiological data regarding hip arthroscopy have been developed. The registries in the United States and Denmark are based on multiple surgical centres, whereas the Swedish registry is based on a single centre [9, 18, 19, 20]. In light of these studies, it is safe to say that registries are functional in collecting and evaluating large amounts of data [19]. Given the absence of a national registry in Italy, the present paper was based on data from the National Hospital Discharge records (SDO) reported by the Italian Ministry of Health, as it represents a valid and reliable tool for the formulation of nationwide epidemiological investigations.

Being aware of the importance of epidemiological data in the assessment and management of hip arthroscopy, this study aims to evaluate the trends in hip arthroscopy procedures in Italy. Demographics, length of hospitalisation (LOS) and diagnosis that led to surgery were compared. Moreover, knowing that the longitudinal examination of national registers is essential to understand the economic burden of hip arthroscopy, the economic impact of the procedure was analysed. Of note is the statistical value of the present study, which is not only useful for future nationwide studies on the Italian population but it is also attractive for an international audience, as it represents a valuable tool for the comparison with international epidemiological data.

## MATERIALS AND METHODS

An investigation of the SDO reported by the Italian Ministry of Health was conducted [12, 13, 14]. The data included information concerning the patient's features (age, sex and domicile), the year when the surgical procedure was performed (from 2001 to 2016), the region where the surgery was made, the length of the hospitalisation, the diagnoses and procedures codes. To calculate the incidence (cases/100,000 residents), population data from the National Institute for Statistics (ISTAT) from each year were used. Hip Arthroscopy was defined by the International Classification of Diseases, Ninth Revision, Clinical Modification (ICD‐9‐CM) primary procedure code: 80.25. Since the aim was to study Hip Arthroscopy in adults, only patients with at least 15 years of age have been selected.

### Statistics

Descriptive statistical analyses including frequency distribution, percentage, mean and standard deviation were performed for data analysis. Continuous data were analysed with mean and standard deviation. Categorical data were presented by frequency and percentage. Incidence rates were calculated using the annual adult population size obtained from ISTAT. The Statistical Package for Social Sciences version 26 (IBM Corp.) and Microsoft Excel (2019) were used for this data analysis.

## RESULTS

### Demographics

**Figure 1:**
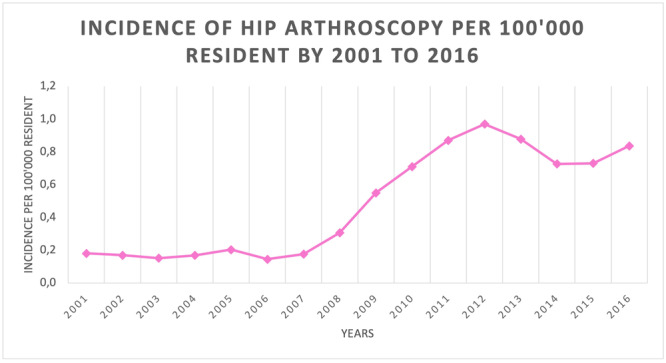
Incidence of hip arthroscopy per 100,000 resident from 2001 to 2016.

Between 2001 and 2016, 4022 Hip Arthroscopy were done in Italy. The incidence during the study period was 0.5 procedures for every 100,000 Italian adult inhabitants. The incidence rate per year ranged from 0.2 in 2001 to 0.8 in 2016, with a peak of 1 in year 2012 (Figure 1).

**Figure 2:**
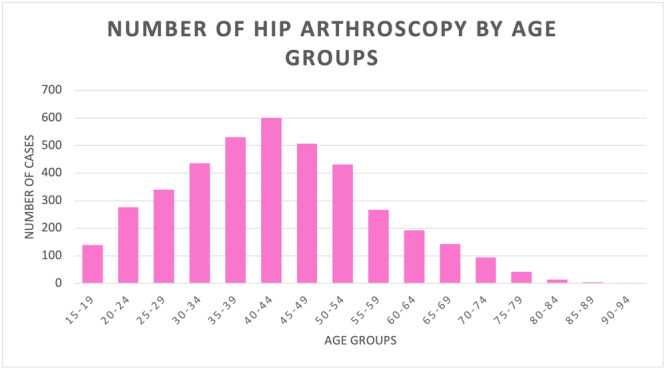
Number of hip arthroscopies by age groups.

During the 16‐year study period, the highest number of procedures was found in the 40‐ to 44‐year age group (Figure 2).

**Figure 3:**
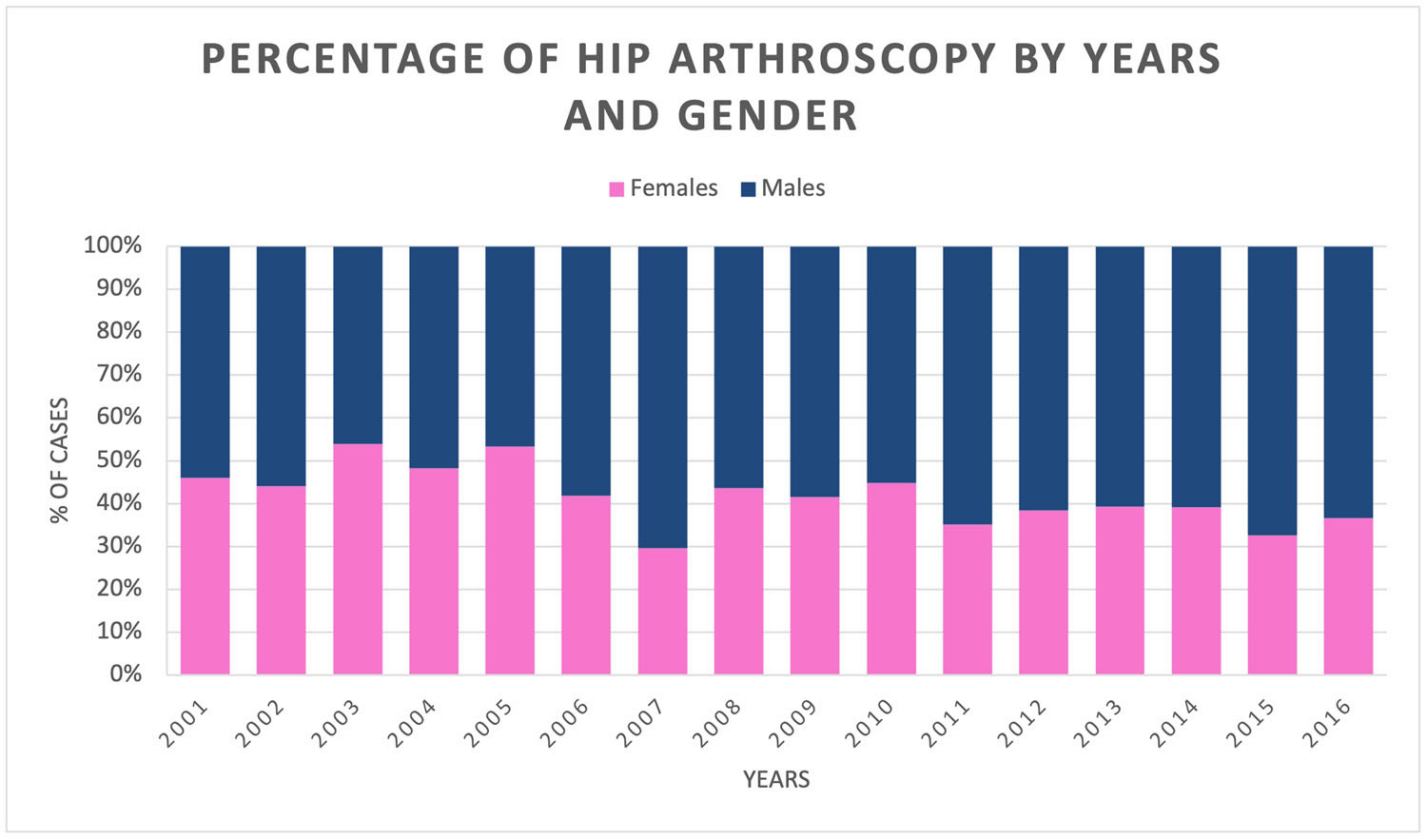
Percentage of hip arthroscopy by years and gender.

Males were more exposed to this surgery than females (females 39.5% and males 60.5%), both in total and over the years (Figure 3).

**Figure 4:**
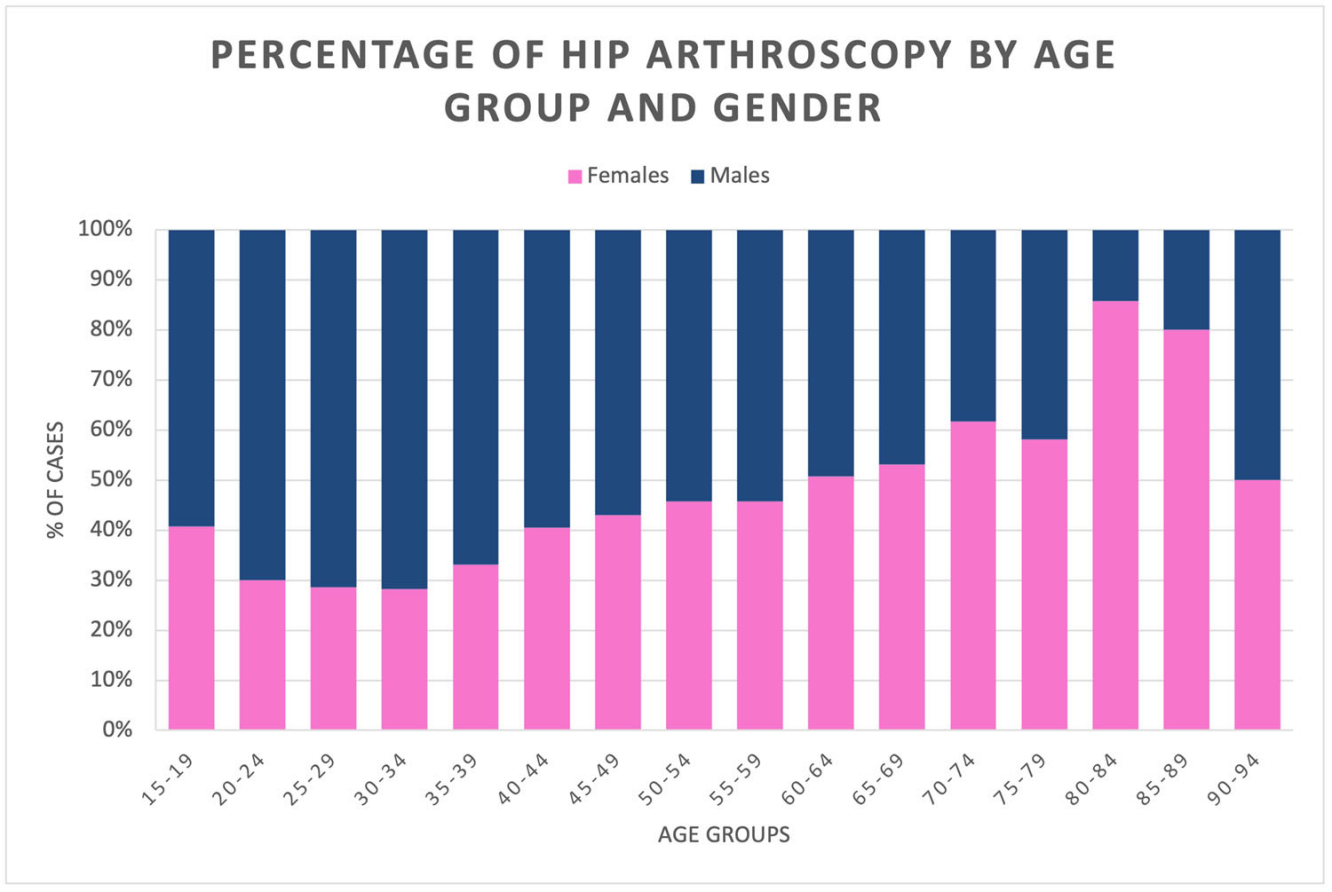
Percentage of hip arthroscopy by age group and gender.

The mean male/female ratio was 1.5. The male/female ratio increased from 1.2 in 2001 to 1.7 in 2016, with a peak of 2.4 in 2007. Up to the age group 55–59, there is a majority of male patients, while from 60 years upward, there is a higher percentage of females (Figure 4).

**Figure 5:**
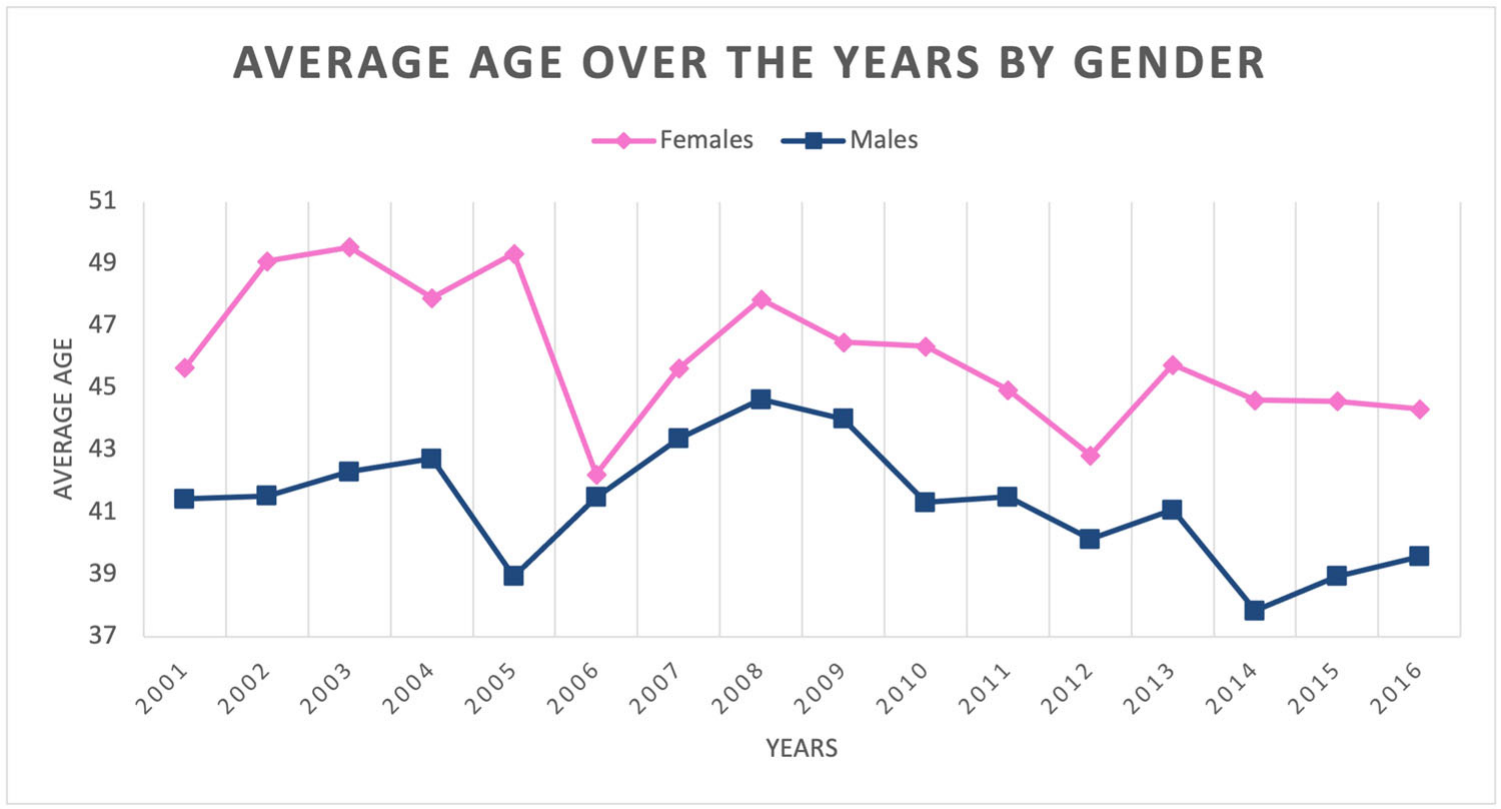
Average age over the years by gender.

From 2001 to 2016, the mean age of patients was 42.6 ± 13.9 (females 45.5 ± 14.6 years and males 40.7 ± 13.2 years). During the entire period, the mean age of females was always higher than that of males (Figure 5).

### Length of the hospitalisation

**Figure 6:**
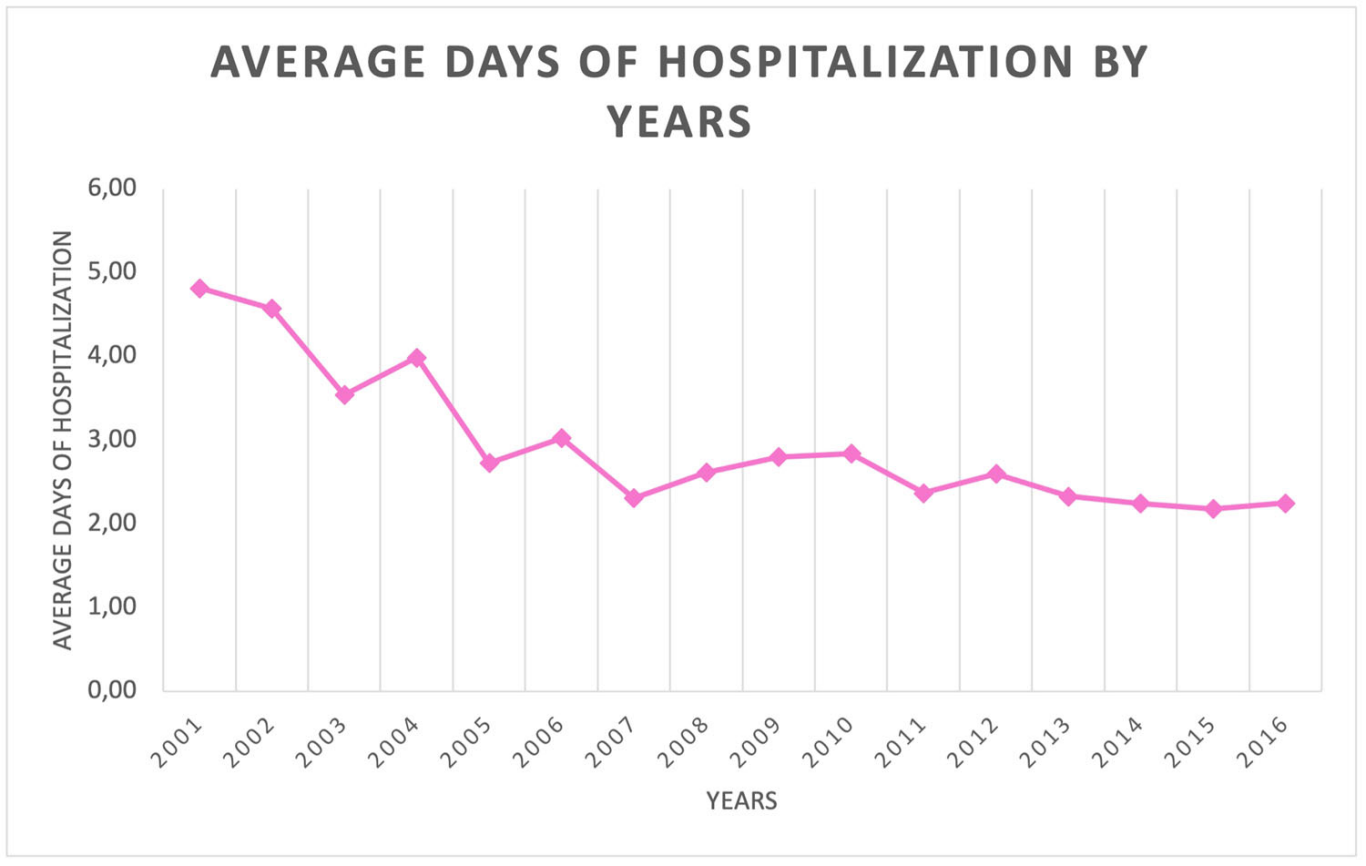
Average days of hospitalisation by years.

The mean LOS was 2.6 ± 3.2 days, with a decreasing trend from 4.8 in 2001 to 2.3 in 2016 (Figure 6).

**Figure 7:**
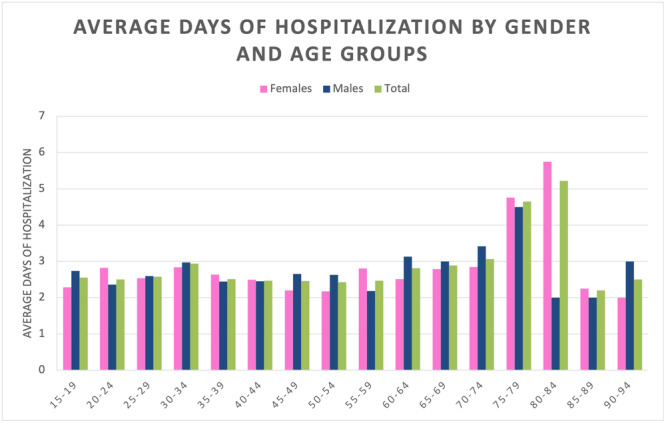
Average days of hospitalisation by gender and age groups.

Overall, patients aged 80–84 years showed more days of hospitalisation (5.2 ± 6.7 days). Men with a greatest number of days of hospitalisation are aged between 75 and 79 years (4.5 ± 6.3 days), while women between 80 and 84 years (5.8 ± 7.2 days; Figure 7).

### Main primary diagnoses

From 2001 to 2016, the main primary diagnoses were pain in joints, pelvic region and thigh (18.9%; diagnosis code: 719.45); osteoarthrosis, localised, primary, pelvic region and thigh (16.6%; diagnosis code: 715.15); other symptoms referable to joint, pelvic region and thigh (14.3%; diagnosis code: 719.65); articular cartilage disorder, pelvic region and thigh (7.9%; diagnosis code: 718.05) and other joint derangement, not elsewhere classified, pelvic region and thigh (7.2%; diagnosis code: 718.85).

### Economic impact

**Figure 8:**
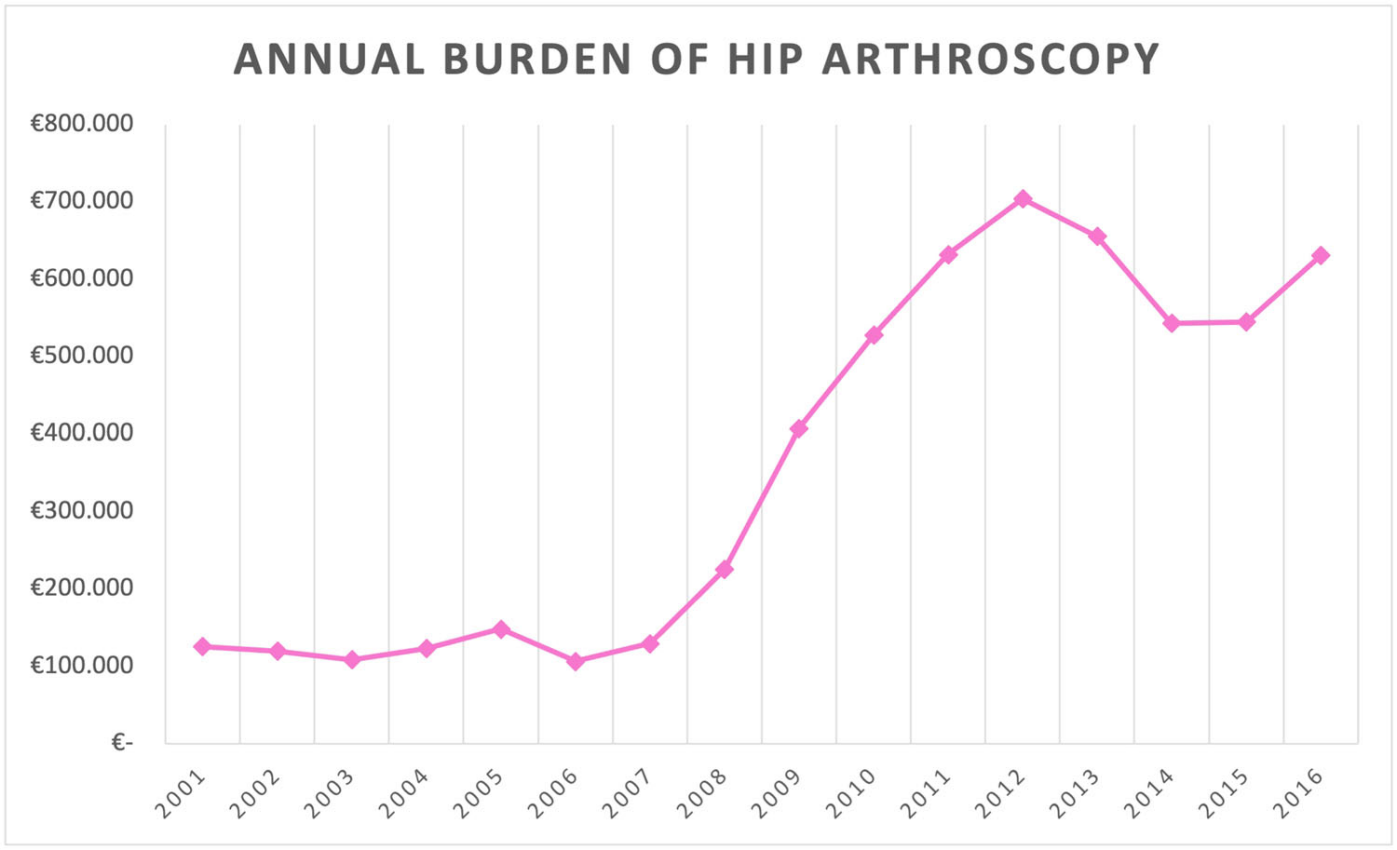
Annual burden of hip arthroscopy.

Every Hip Arthroscopy procedure had a mean Italian hospital reimbursement from 1361€ (more than 1‐day stay, with an increase of 7€ for each additional day of hospitalisation) to 1512€ (1‐day stay treatment). During the 16‐years period, a total cost of 5,733,062€ has been calculated. Hip Arthroscopy procedures cost about 358,316€ ± 239,818€ each year in Italy, with costs ranging from 106,445€ in 2006 to 704,030€ in 2012 (Figure 8).

## DISCUSSION

Numerous studies describing the epidemiology of hip arthroscopy are found in the literature but, to our knowledge, this 16‐year nationwide registry study is the first in‐depth, national assessment of hip arthroscopy procedures ever carried out in Italy. The present study not only sets the ground for future Italian nationwide studies, but it also lays the basis for the development of epidemiological investigations on the European population, extending its statistical value to an international audience. The national register, which is compiled through a compulsory reporting system for the entire population, provided all the data on dates, diagnoses and number of hip arthroscopy procedures conducted throughout the study period. The data of this study shows a net increase in the population‐weighted incidence rate for hip arthroscopy procedures performed in Italy between the years 2001 and 2016, suggesting the growing relevance of hip arthroscopy in the healthcare system. Nonetheless, the comparison of the results of this study with those of the literature is challenging. Three studies [9, 18, 20] analysed the demographics and the development of national registries of Sweden, Great Britain and Denmark, evaluating a shorter period of time and without showing the variation in the number of interventions carried out every year. In particular, the intervals assessed by each were between 2011 and 2013 for the Swedish [20], 2013 and 2015 for the British [9] and 2012 and 2015 for the Danish [18] papers. Two studies [6, 19] considered a larger time span, demonstrating only four yearly variations in the number of hip arthroscopies (from 2021 to 2015, 2016, 2017, 2018) in one case [19] and none in the other [6], not allowing a complete and adequate comparison with the results of the present study. Only Kremers et al. [16] documented that the utilisation of hip arthroscopy procedures in the United States increased every year from 2005 to 2013, reaching an incidence of 16.7 per 100,000 in 2013. In light of this, further long‐term studies might be useful in the assessment of yearly trends and variations of hip arthroscopy in the population.

When examining age and sex, the findings of this paper were inconsistent with the literature. During the 16‐year period of this study, males were more exposed to the surgery than females. Three papers [9, 16, 19] described the opposite, as more females underwent surgery during the study period compared to males. Contrarily, Sansone et al. [20] and Disegni et al. [6] are in line with the present study, having more male (67% and 55%, respectively) than female (33% and 45%, respectively) patients undergoing hip arthroscopy procedures. This is inconsistent with the female predominance outlined in previous hip arthroscopy cohorts [15, 21]. The data of the present investigation shows that the mean age of women was always higher than that of men. While the results from an epidemiological analysis of hip arthroscopy in France conducted by Disegni et al. [6] is in line, a second paper [4] reported a higher mean age in men than in women. The discrepancies found between the findings of this study and those of the literature clearly explain how healthcare systems and care practices differ from one country to an other and how a study based solely on the Italian population is needed. Moreover, the data available for the conduction of the present study did not allow us to carry out an in‐depth analysis of the reasons behind such inconsistencies.

The mean LOS decreased over the 16‐year study period. The reported LOS reduction could be attributable to better perioperative care, but it could also be a consequence of the efforts made by the hospitals in the attempt to reduce economic losses [22].

To our knowledge, no article evaluating the costs of hip arthroscopy has been published. In order to allow a comparison with future papers, annual costs of the operation were estimated. Trends of the annual costs of hip arthroscopy procedures observed during the study period are in line with those of the incidence rate, ranging from a minimum value registered in 2006 and a maximum value in 2012. Several studies prove the cost‐effectiveness of hip arthroscopy, analysing healthcare resource usage and comparing costs of surgical and nonsurgical approaches for the treatment of common hip pathologies like femoroacetabular impingement [3, 5, 8]. However, further nation‐based studies are needed to assess the impact that hip arthroscopy has on the national healthcare system and health economics in general.

This study has several strengths, in particular the inclusion of a large population living in the same national territory over a relatively long period of time. It emphasises the significance of a comprehensive clinical documentation, as it was entirely based on a worthwhile and reliable population‐based registry. Through the application of an accurate database, it is possible to improve the clinical relevance and the efficiency of administrative codes related not only to hip arthroscopy but to all the procedures [2].

### Limitations

The data for the present research was sourced directly from the Italian Ministry of Health. Both public and private hospitals in Italy must regularly provide SDO to the ministry. It is important to mention that the ministry does not release these records to the public on an annual basis. Consequently, the data reported in this study reflects the latest available records within the constraints of the Italian health information system. Furthermore, additional limitations should be noted. First, since the study is based on administrative databases containing ICD‐9 codes from different regions and hospitals, clinical and surgical details were not available. Therefore, it was not possible to evaluate specific diagnosis and examine their trends and outcomes, as well as their complications. Additionally, the laterality of the operations could not be confirmed due to unavailable data. Second, due to the use of nonspecific diagnosis codes, the ability to determine the type(s) of procedures performed (i.e., femoroacetabular impingement, labral repairs) or the underlying conditions was highly restricted. Finally, patients are not registered with a unique ID number. As a result, it was impossible to differentiate between operations performed in the same patient or bilateral procedures and to track the same individual across multiple surgeries. This discrepancy could lead to an underestimation of the results.

## CONCLUSIONS

The present study is the first comprehensive epidemiological analysis of hip arthroscopy ever conducted across the entire Italian population. Hip arthroscopy procedures have increased substantially between the years of the study, as well as the costs related to the surgery, with males being more exposed than females. Additionally, the mean LOS was found to have decreased over the 16‐year study period. The findings of this study constitute valuable clinical and scientific evidence for the Italian healthcare system useful not only for the formulation and improvement of national healthcare policies but also to improve awareness and accessibility of hip arthroscopy. Furthermore, epidemiological studies like this may have a role in the comparison of national surgical trends with those of other countries.

## AUTHOR CONTRIBUTIONS

**Umile Giuseppe Longo**, **Sergio De Salvatore**, **Giovanni Intermesoli**: Manuscript preparation; study design; database interpretation; manuscript revision. **Umile Giuseppe Longo**, **Ilaria Piergentili**, **Rocco Papalia**: Manuscript preparation; database interpretation; statistical analysis. **Giovanni Intermesoli**, **Ilaria Piergentili**, **Sergio De Salvatore**: Manuscript preparation; figures and tables preparation; study design. **Umile Giuseppe Longo**, **Stefano Zaffagnini**, **Vincenzo Denaro**, **Kristian Samuelsson**: Manuscript preparation; database interpretation. **Umile Giuseppe Longo**, **Giovanni Intermesoli**, **Vincenzo Denaro**, **Rocco Papalia**, **Pieter D'Hooghe**: Study design; manuscript revision. The authors read and approved the final manuscript.

## CONFLICT OF INTEREST STATEMENT

The authors declare no conflict of interest.

## ETHICS STATEMENT

The study was conducted in accordance with the Declaration of Helsinki and approved by the Institutional Review Board (or Ethics Committee) of Fondazione Policlinico Universitario Campus Bio‐Medico di Roma.

## Data Availability

The data sets used and/or analysed during the current study are not publicly available due on our policy statement of sharing clinical data only on request but are available from the corresponding author on reasonable request. The access to the database is on request. All data were obtained by the Direzione Generale della Programmazione Sanitaria—Banca Dati SDO of the Italian Ministry of Health.
